# The role of funders: Wellcome Trust

**DOI:** 10.1186/s40985-016-0022-7

**Published:** 2016-08-24

**Authors:** Sarah Molton

**Affiliations:** Our Planet, Our Health, Wellcome Trust, London, UK

**Keywords:** Global Health, Planetary health, Environment, Transdisciplinary research, Foundations

## Abstract

Wellcome exists to improve health for everyone by helping great ideas to thrive. We are a global charitable foundation, both politically and financially independent. We support scientists and researchers take on big problems, fuel imaginations and spark debate. The health of the global population and the planet are inextricably linked but there is a poor ecological fit between what we are asking of the planet and its resilience. If the complex natural systems we rely on for clean air, fresh water, fertile soil, biodiversity and a stable climate are threatened, so too is our health. The challenge is to secure the health and well-being of present and future generations whilst responsibly stewarding the planet. As research continues to unravel our understanding of the vital links between health and the environment, we become better equipped to develop robust, coherent and coordinated solutions that jointly reduce threats to human health and to the surrounding environment that sustains it. There are already clear opportunities for change but more research is needed.

Our Planet, Our Health was identified as new priority area for Wellcome in 2015. We support work that embraces and stimulates creative partnerships, collaborating across disciplines and sectors, because we believe that we need a diversity of competencies to tackle these complex problems. Our aim is to gain deeper insights into these issues, to inform the global response through transdisciplinary research and develop policies that will help mitigate the risks to human health.

## Main text

The Wellcome Trust is an independent global charitable foundation dedicated to improving health. Since 1936, our funding has helped to save millions of lives worldwide, through science, research and engagement with society. This support includes transformative work like the sequencing of the human genome, and research that established the front-line drugs for malaria.

As a funder of health research, we need to understand how health and the environment interact. Just as *The Lancet* as a leading health journal is addressing the connections between health and the environment with the Planetary Health Commission, we as a funder need to consider these links carefully. Increasing population growth, combined with changes in consumption, is testing the resilience of the planet on which we live. This ecological overreach is threatening prosperity, health and well-being for current and for future generations.

For three and a half years, a team at Wellcome has been striving to build our understanding of these global challenges and works out what role Wellcome can play in developing the field of planetary health and safeguarding the health of our planet and its inhabitants.

In 2013, we were allocated a small amount of research funding and used this to fund a range of pilot projects. When we put out a call for proposals, we had around 840 expressions of interest from researchers around the world, from ecologists and epidemiologists to engineers, all of them thinking in a transdisciplinary way about research questions that bridge environment and health. It is clear there is an appetite for new research opportunities, both from the academic community and from policy makers who need evidence, and Wellcome can play a part in creating and funding these opportunities.

The next step in Wellcome’s investment in this area is Our Planet, Our Health, an initiative, announced in September 2015, which includes a commitment to invest £75 million in the area of planetary health over the next 5 years. Through Our Planet, Our Health, we will provide funding for international research programmes, develop strategic partnerships and, importantly, engage with society.

From working with partners and talking to others in the field, we are convinced of the importance of evidence, education and engagement in furthering the development of planetary health and ensuring it has an impact on human health worldwide.

Wellcome’s philosophy is that good health makes life better. We work to improve health for everyone by helping great ideas to thrive; the planetary health community is full of people with great ideas and the passion to bring about meaningful change, and Wellcome is committed to working with them.

